# Biomarkers and Emerging Approaches in Gastroenteropancreatic Neuroendocrine Neoplasms (GEP-NEN): Current Evidence and Future Directions

**DOI:** 10.3390/cancers18182935

**Published:** 2026-09-10

**Authors:** Gabriella D’Orazi, Laura Monteonofrio, Alessandra Verdina, Alessia Garufi

**Affiliations:** 1 Departmental Faculty of Medicine, UniCamillus-Saint Camillus International University of Health Sciences, 00131 Rome, Italy; 2 Unit of Cellular Networks and Molecular Therapeutic Targets, Department of Research and Advanced Technologies, IRCCS Regina Elena National Cancer Institute, 00144 Rome, Italy; laura.monteonofrio@ifo.it (L.M.); alessandra.verdina@ifo.it (A.V.)

**Keywords:** gastroenteropancreatic neuroendocrine neoplasms (GEP-NEN), biomarkers, liquid biopsy, spatial transcriptomics, NETest, precision oncology, artificial intelligence (AI), machine learning (ML), radiomics

## Abstract

Gastroenteropancreatic neuroendocrine neoplasms (GEP-NENs) comprise a biologically and clinically heterogeneous spectrum of tumors with substantial inter- and intratumoral variability. Despite advances in molecular classification, imaging, and treatment, accurate diagnosis, prognostication, and disease monitoring remain challenging. Established biomarkers, including chromogranin A (CgA) and 5-hydroxyindoleacetic acid (5-HIAA), provide limited and context-dependent information, highlighting the need for more biologically informative approaches. Recent advances in liquid biopsy, transcriptomic and genomic profiling, spatial technologies, molecular imaging, radiomics, and artificial intelligence are expanding the ability to characterize tumor biology and evolution. However, biological association and analytical validity do not necessarily translate into clinical utility. This narrative review critically evaluates established and emerging biomarkers according to the biological information they provide, with particular emphasis on molecular heterogeneity, tumor evolution, and the distinction between validated and investigational applications. We further discuss how complementary molecular, circulating, spatial, imaging, and clinical information may be integrated through multimodal and artificial intelligence-based approaches to advance tumor characterization and support precision oncology in GEP-NENs.

## Simple Summary

Gastroenteropancreatic neuroendocrine neoplasms (GEP-NENs) are a diverse group of tumors that can behave very differently from one another. Blood tests and other biomarkers may help doctors understand tumor biology, estimate prognosis, and monitor patients during treatment. However, currently available biomarkers have important limitations and no single test can capture all the information needed to guide patient care. Emerging approaches, including analysis of circulating tumor DNA and cells, imaging features, and molecular profiles, may offer additional insights into these tumors. Integrating complementary molecular, circulating, imaging, and clinical information through multimodal approaches and artificial intelligence-based models may improve tumor characterization and generate clinically relevant hypotheses, although their impact on patient management remains to be established.

## 1. Introduction

Neuroendocrine neoplasms (NENs) account for 0.5% of all malignancies, and 62–70% of these are found in the gastroenteropancreatic system. Gastroenteropancreatic neuroendocrine neoplasms (GEP-NENs) originate from neuroendocrine cells of the diffuse endocrine system of the gastrointestinal tract and pancreatic islets [1]. Although historically considered rare, the reported incidence of GEP-NENs has increased substantially over recent decades, likely reflecting, at least in part, improvements in diagnostic imaging, pathological classification, and clinical awareness [2,3]. According to the 2022 World Health Organization (WHO) classification, GEP-NENs comprise well-differentiated neuroendocrine tumors (NETs) including pancreatic, gastric, small bowel, and rectal NETs and poorly differentiated neuroendocrine carcinomas (NECs) [4]. Tumor grade is based on mitotic count and Ki-67 proliferation index and includes G1, G2, and G3 categories, whereas NECs are, by definition, poorly differentiated, high-grade neoplasms [4,5].

However, substantial biological heterogeneity persists within each category, limiting the predictive accuracy of morphology alone. Consequently, conventional histopathological classification may be insufficient to accurately predict tumor behavior, treatment response, and clinical outcome [6]. Importantly, this heterogeneity extends beyond differences between anatomical sites and histological categories. GEP-NENs display intertumoral, intratumoral, and temporal heterogeneity, with molecular alterations, transcriptional states, receptor expression, proliferative activity, and tumor–microenvironment interactions varying within individual tumors and evolving during disease progression or treatment [7,8]. This complexity provides a rationale for evaluating complementary biomarker layers beyond conventional single-analyte approaches [9].

Although numerous circulating and tissue-based candidate biomarkers have been proposed over the last decade, only a limited number have demonstrated sufficient evidence to support their use in routine clinical practice [10,11]. A critical distinction should therefore be made between biological association, indicating an association with a tumor phenotype or biological process; analytical validity, referring to assay accuracy and reproducibility; clinical validity, referring to the ability to discriminate or predict clinically relevant states; and clinical utility, requiring evidence that biomarker-guided use improves clinical decision-making or patient outcomes [12]. Many emerging GEP-NEN biomarkers remain supported primarily by biological associations and clinical validity data, whereas evidence demonstrating clinical utility remains limited [11].

**Figure 1:**
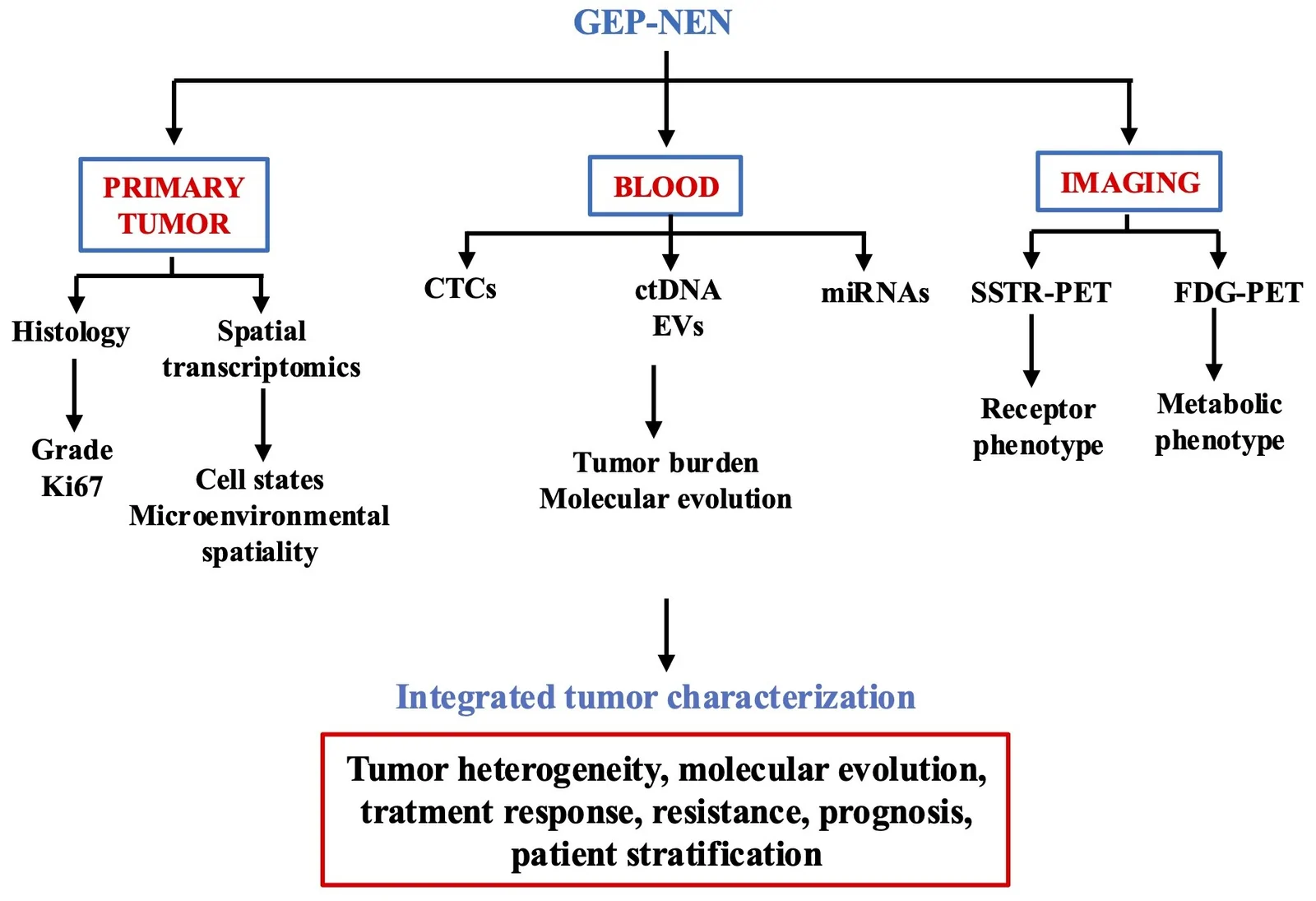
Translational framework of established and emerging GEP-NENs biomarkers: from biological signals to clinical utility.

Rather than providing a conventional catalogue of candidate biomarkers, this review proposes a translational framework that considers established and emerging biomarkers GEP-NENs according to the biological information they capture, their clinical validity, and, critically, the level of evidence supporting their clinical utility (Figure 1). We examine the biological layers interrogated by different biomarker approaches, their complementarity with conventional pathology and imaging, and their potential relevance to precision oncology. Throughout the review, guideline recommendations are reported within the relevant biomarker-specific sections and summarized in the “Guideline status” columns of Table 1, Table 2 and Table 3, with a distinction between biomarkers supported for selected clinical applications and those that remain investigational. Given the molecular and translational scope of this review, we do not propose a clinical decision-making algorithm for biomarker selection, which would be premature for several emerging biomarker classes, instead, we provide a context-dependent appraisal of established and emerging biomarkers according to the clinical settings in which they have been investigated and the level of evidence supporting their clinical validity and utility.

A literature search was performed to identify studies addressing established and emerging biomarkers and biomarker technologies in GEP-NENs. A PubMed/MEDLINE search was performed covering approximately the last 15 years using combinations of terms related to “GEP-NEN,” “neuroendocrine tumor,” “neuroendocrine carcinoma,” “biomarker,” “chromogranin A,” “5-HIAA,” “circulating tumor cells,” “circulating tumor DNA,” “microRNA,” “extracellular vesicles,” “NETest,” “molecular imaging,” “radiomics,” “spatial transcriptomics,” “artificial intelligence”, and “multimodal integration.” Additional relevant publications were identified through reference-list screening and citation tracking. The literature was considered according to its relevance to GEP-NEN biology, biomarker performance, clinical validation, clinical utility, and translational potential, with particular attention to prospective studies, independent validation cohorts, clinical guidelines, systematic reviews, and recent high-quality studies. Given the narrative nature of the review, no formal systematic-review protocol or quantitative meta-analysis was undertaken.

## 2. Limitations of Current Clinical, Histopathological, and Molecular Assessment

GEP-NENs exhibit a broad spectrum of clinical presentations and are broadly classified as functional or non-functional tumors according to the presence or absence of hormone-related clinical syndromes [13]. Functional GEP-NENs secrete biologically active hormones responsible for characteristic clinical manifestations whereas non-functional GEP-NENs lack a hormone-related clinical syndrome and are often diagnosed incidentally or during investigations of tumor-related symptoms [13].

Histopathological evaluation remains the diagnostic gold standard for GEP-NENs, based on biopsy or surgical resection specimens [4]. Diagnosis relies on hematoxylin and eosin examination followed by immunohistochemical (IHC) staining of neuroendocrine differentiation markers particularly chromogranin A (CgA), synaptophysin, and, when appropriate, insulinoma-associated protein 1 (INSM1), together with Ki-67 assessment for grading [14]. However, conventional histopathological assessment has several limitations, including sampling bias, intratumoral heterogeneity, diagnostic overlap between high-grade NETs and NECs, and the inability of Ki-67 alone to fully capture the biological complexity underlying tumor differentiation and grade. Thus, well-differentiated NETs typically show diffuse CgA and synaptophysin expression, whereas poorly differentiated NECs often retain synaptophysin but show reduced or heterogeneous CgA expression [14].

The limitations of preoperative tissue sampling were supported by a systematic review and meta-analysis of 26 studies including 864 patients with pancreatic NETs (PanNETs), which reported an overall concordance of 80.3% between EUS-guided and surgical grading, with undergrading occurring more frequently than overgrading. Individual data analysis showed that among nonconcordant patients, the median Ki-67 difference was 3%, highlighting the potential impact of sampling-related limitations on preoperative risk stratification [15]. These limitations are particularly relevant in the context of the marked spatial and temporal heterogeneity of GEP-NENs. A biopsy may capture only a limited proliferative or molecular compartment of a heterogeneous tumor, while metastatic lesions may harbor distinct molecular profiles, as demonstrated in PanNET [16].

Molecular characterization therefore requires careful distinction between well-differentiated NETs and poorly differentiated NECs, as the spectrum and biological relevance of genomic alterations varies according to tumor site and differentiation. In several GEP-NEN subtypes, loss-of-function alterations predominate over activating molecular events. According to the European Guidelines, molecular stratification using alterations in *MEN1* (multiple endocrine neoplasia type 1), *DAXX*/*ATRX* (death domain-associated protein, alpha-thalassemia/mental retardation syndrome X-linked), *TP53*, and *RB1* (retinoblastoma) genes may provide additional biological and diagnostic information, particularly for distinguishing high-grade NETs from NECs [17]. Alterations involving *MEN1*, *DAXX*, and *ATRX* are particularly relevant to PanNET biology, whereas *TP53* and *RB1* alterations are more characteristic of poorly differentiated NECs [17]. A seminal whole-exome study of 68 sporadic PanNETs identified somatic mutations of *MEN1* and *ATRX*/*DAXX* in, respectively, 44% and 43% of tumors, as well as alterations in the *mTOR* (mammalian target of rapamycin) pathway in 14% of analysed tumors [18]. Mutations in *MEN1*, *DAXX*/*ATRX* were associated with prolonged survival compared to patients whose tumors lacked these mutations. In addition, alterations in *mTOR* pathway genes were suggested to potentially be used to stratify patients for treatment with mTOR inhibitors [18]. Subsequent studies linked *MEN1* mutations to tumor initiation in PanNETs and loss of *ATRX*/*DAXX* expression to chromosomal instability, activation of the alternative lengthening of telomeres (ALT) pathway, and adverse clinical outcomes, including metastatic disease and shorter disease-free survival [19,20]. ALT has also emerged as a potential molecular biomarker in PanNETs, although its integration into routine clinical practice remains under investigation [21].

Taken together, these findings illustrate that both histopathological and molecular assessment provide essential but incomplete representations of GEP-NEN biology. Although molecular alterations may offer biologically informative and, in selected settings, diagnostically relevant information, their routine use as predictive or treatment-selection biomarkers remains limited. Accordingly, circulating and spatial approaches should be considered complementary to tissue-based assessment, potentially providing additional information on tumor heterogeneity and molecular evolution rather than simply replacing conventional tissue diagnosis [8].

## 3. Classical Monoanalyte Biomarkers

The limitations of conventional histopathological and molecular assessment have increased interest in circulating biomarkers that may provide complementary information through minimally invasive approaches. Among these, classical circulating monoanalyte biomarkers, including CgA, 5-hydroxyindoleacetic acid (5-HIAA), neuron-specific enolase (NSE), and pancreastatin, remain widely used in the clinical management of GEP-NENs, particularly for biochemical characterization and disease monitoring [22]. However, their clinical performance is influenced by tumor subtype, functional status, tumor burden, and other biological and clinical factors (Table 1). Moreover, their ability to capture molecular heterogeneity and the dynamic evolution of GEP-NENs is inherently limited, providing a rationale for the development of more comprehensive biomarker approaches. Their interpretation may also be affected by non-tumor-related factors, differences in assay methodology, and lack of assay standardization, which can limit comparability across studies and clinical settings and reduce analytical reproducibility [23]. These limitations highlight the need for biomarker strategies that integrate multiple biological signals rather than relying on a single circulating analyte.

### 3.1. Chromogranin A (CgA)

Chromogranin A (CgA) is an acidic glycoprotein present in the secretory granules of endocrine and neuroendocrine cells and is one of the most widely used circulating biomarkers in GEP-NENs [24]. However, its low specificity, variable sensitivity, and imperfect correlation with tumor burden limit its use as a stand-alone biomarker [24]. CgA levels are generally higher in well-differentiated NETs than in poorly differentiated NEC [25], and may be increased in functional and metastatic disease [26]. Evidence from the RADIANT-2 and RADIANT-3 trials further illustrates the distinction between prognostic and predictive value. RADIANT-2 included patients with advanced (unresectable or metastatic), well- or moderately differentiated NETs and a history of symptoms attributed to carcinoid syndrome (CS) [27]; whereas RADIANT-3 included patients with advanced well- or moderately differentiated PanNETs (G1–G2) [28]. In these analyses, elevated baseline CgA was associated with poorer overall survival, supporting its prognostic value. Importantly, in the RADIANT-2 trial, the clinical benefit associated with everolimus treatment was independent of baseline CgA levels, indicating that elevated CgA identified patients with a poorer prognosis but did not predict differential treatment efficacy [27]. Similarly, in patients treated with peptide receptor radionuclide therapy (PRRT), circulating CgA levels showed limited ability to predict treatment response [29].

The clinical interpretation of CgA is strongly site- and context-dependent [30]. Its circulating concentration can be substantially affected by non-tumor-related factors, including proton pump inhibitor (PPI) therapy [31], renal dysfunction [32], gastrointestinal conditions and other non-tumor related factors [30,33], which may lead to falsely elevated CgA levels and substantially reduce its diagnostic specificity in real-world settings [34]. Differences in assay methodology and the lack of assay standardization may further limit the comparability and reproducibility of CgA measurements across clinical settings. Accordingly, CgA should be regarded primarily as a marker of neuroendocrine secretory phenotype rather than as a universal diagnostic biomarker. Consistent with these limitations, ENETS (European Neuroendocrine Tumor Society) guidance for small-intestinal (SI)-NETs considers circulating biomarkers useful for diagnosis, follow-up, and treatment monitoring in selected patients, while specifically recommending against the use of CgA for screening because of the risk of false-positive elevations [35]. Similarly, ENETS guidance for non-functioning PanNETs states that no routine circulating biomarker is currently established for diagnosis, although CgA may have a role in disease monitoring when measurements are performed consistently using the same assay [36]. Overall, CgA may provide useful biological and prognostic information in selected clinical contexts, but its limited tumor specificity and dependence on clinical and analytical context preclude its use as a universal diagnostic or predictive biomarker (Table 1). CgA should therefore be interpreted within the broader clinical context rather than used in isolation [37].

### 3.2. 5-Hydroxyindoleacetic Acid (5-HIAA)

5-Hydroxyindoleacetic acid (5-HIAA), the major urinary metabolite of serotonin, is another commonly used biochemical marker, particularly in patients with CS [38]. Its clinical utility is particularly relevant in a small percentage of GEP-NENs, corresponding to serotonin-secreting tumors associated with CS (about 15–20%) [38]. 5-HIAA is more commonly measured in 24 h urine collections, although plasma measurements are also available [39]. Elevated urinary 5-HIAA has a sensitivity of approximately 70% and a specificity of 90–100% in patients with CS, whereas, in the absence of CS, its sensitivity decreases to approximately 35% [38]. Although baseline urinary 5-HIAA has shown limited prognostic value, longitudinal changes may provide additional information in selected patients; in a study of patients with NETs, a shorter 24 h urinary 5-HIAA doubling time was associated with an increased risk of disease progression and disease-specific mortality [39]. Several non-neoplastic conditions, including frequent consumption of serotonin-rich food or dopamine-rich foods/medications, and malabsorption syndromes, may increase urinary 5-HIAA levels, thereby reducing its diagnostic specificity [38]. Its biological specificity for serotonin-producing tumors is an advantage, but this same specificity limits its applicability across the heterogeneous GEP-NEN spectrum. It is therefore particularly informative in serotonin-secreting SI-NETs and in patients with CS rather than as a universal biomarker across GEP-NENs. Current ENETS guidance for SI-NETs supports 5-HIAA measurement in patients with suspected CS and for treatment monitoring in patients with established CS, illustrating how biomarker value depends strongly on tumor phenotype and clinical context rather than on the umbrella diagnosis of GEP-NEN [35]. Consistent with these limitations, a study of 371 patients with gastrointestinal (GI)-NETs demonstrated that urinary 5-HIAA levels had limited prognostic value for overall survival (OS) [40]. Thus, although 5-HIAA remains an established biomarker in the appropriate clinical setting, its utility is largely context-dependent and is limited outside serotonin-secreting tumors associated with CS (Table 1).

### 3.3. Neuron-Specific Enolase (NSE) and Pancreastatin

Neuron-specific enolase (NSE) is a neuron-specific isoform of the glycolytic enzyme enolase that is physiologically expressed in neurons and neuroendocrine cells [41]. While CgA is more commonly used in well-differentiated NETs, NSE may provide complementary information, particularly in high-grade and poorly differentiated GEP-NENs, especially NECs [42]. Elevated pretreatment serum NSE levels have been associated with poorer prognosis, including shorter OS and more rapid disease progression [43]. In digestive NEC, both CgA and NSE have been reported to be elevated in approximately 60% of patients, and retrospective data suggest that levels exceeding twice the upper limit of normal may have prognostic value; however, their clinical utility requires confirmation in prospective studies [44]. More recently, serial NSE measurements have been investigated as a potential tool for monitoring treatment response in digestive NECs; in an exploratory analysis of JCOG1213, a decline in NSE during chemotherapy was associated with treatment response, although its routine clinical use remains to be established [45]. Overall, NSE may have greater biological and prognostic relevance in high-grade or poorly differentiated GEP-NENs, particularly NECs, than in low-grade, well-differentiated NETs. However, its limited specificity and the lack of prospective validation restrict its routine clinical implementation (Table 1).

Pancreastatin is a peptide derived from the proteolytic processing of CgA and has been investigated as a potential prognostic biomarker [46]. Its role as a stand-alone circulating biomarker remains insufficiently established. In a study including patients with small-bowel and PanNETs, elevated preoperative pancreastatin levels were significantly associated with shorter progression-free and OS and remained independently associated with poorer outcomes after multivariate adjustment [46]. However, the available evidence remains insufficient to establish pancreastatin as a validated biomarker for routine clinical decision-making in GEP-NEN (Table 1).

## 4. Liquid Biopsy Approaches: Circulating Tumor Cells (CTCs), Circulating Tumor DNA (ctDNA), MicroRNAs (miRNAs), and Extracellular Vesicles (EV)

Liquid biopsy is a sampling strategy capable of interrogating several biological layers. It has been best studied in blood, although it can be performed in other body fluids such as urine or cerebrospinal fluid. Its mechanism is based on the detection of circulating tumor components, such as circulating tumor cells (CTCs), circulating tumor DNA (ctDNA), circulating non-coding RNA and microRNA (miRNAs), and extracellular vesicles (EVs), each reflecting different aspects of tumor biology [47,48]. The principal conceptual advantage of liquid biopsy is therefore its potential to provide longitudinal and spatially complementary information that may help overcome some of the limitations associated with conventional tissue-based assessment. Liquid biopsy holds potential for managing neuroendocrine neoplasms [49].

### 4.1. Circulating Tumor Cells (CTCs)

Circulating Tumor Cells (CTCs) represent a minimally invasive cellular biomarker of tumor dissemination and metastatic potential [50]. The CellSearch system is a standardized platform for CTCs enumeration based on immunomagnetic capture using antibodies against epithelial cell adhesion molecule (EpCAM). Although CellSearch has received U.S. Food and Drug Administration (FDA) clearance for CTC enumeration in specific metastatic breast, colorectal, and prostate cancer settings [51], its application to GEP-NENs remains investigational and is not an established clinical indication. An important molecular limitation of conventional CTC isolation is the selection bias introduced by epithelial-marker-dependent enrichment. During epithelial-to-mesenchymal transition (EMT), tumor cells may downregulate epithelial markers such as EpCAM and acquire mesenchymal characteristics, including increased expression of vimentin and N-cadherin [52]. Consequently, CTC populations captured by EpCAM-dependent systems may not represent the full spectrum of circulating tumor cells and may underrepresent cells displaying EMT-related phenotypes [53,54]. Alternative CTC enrichment and detection strategies may partially address this limitation, including size-based filtration approaches such as ISET, microfluidic platforms exploiting cell size or deformability, label-free physical separation. Multi-marker strategies incorporating mesenchymal antigens or antibody cocktails may also improve the detection of CTC subpopulations that are poorly captured by EpCAM-dependent methods. However, these approaches remain heterogeneous and insufficiently standardized in GEP-NENs, and their comparative clinical value has not yet been established. The feasibility of detecting CTCs in patients with metastatic NETs using the CellSearch platform was demonstrated in a study in which CTCs were detected in 43% of patients with midgut NETs and 21% of patients with PanNETs [53]. CTCs count in peripheral blood was associated with disease progression and serum CgA levels, although their relationship with tumor grade appears less consistent [53]. CTCs have also been investigated as dynamic biomarkers for treatment monitoring. In a study including 138 patients with metastatic NETs, early changes in CTCs count after treatment were associated with treatment response and survival outcomes. Patients who maintained undetectable CTCs or achieved a ≥50% reduction from baseline experienced more favorable progression-free and OS than those with persistently elevated or increasing CTCs count, suggesting that early CTCs dynamics may provide prognostic information during treatment [55]. In the exploratory CALM-NET trial, conducted in patients with functional, well- or moderately differentiated midgut NETs treated with lanreotide, the absence of detectable CTCs at baseline was associated with a numerically higher probability of symptomatic response to somatostatin analogue (SSA) therapy, although this association did not reach statistical significance [56]. In GEP-NENs, the hormone somatostatin, along with its synthetic counterparts somatostatin analogs (SSAs), is fundamentally important because well-differentiated GEP-NEN cells abundantly overexpress somatostatin receptors (SSTRs), particularly SSTR2 and SSTR5 [57].

Despite these encouraging findings, several challenges currently preclude the routine clinical use of CTCs in GEP-NENs (Table 2). Detection sensitivity remains low, particularly in well-differentiated and low-grade tumors, EpCAM-based enrichment may fail to capture cells undergoing EMT, standardized detection platforms beyond CellSearch are lacking, and prospective multicenter validation remains limited.

### 4.2. Circulating Tumor DNA (ctDNA)

Circulating tumor DNA (ctDNA) is an emerging liquid biopsy biomarker consisting of fragmented DNA released into the bloodstream by tumor cells from primary or metastatic lesions. Unlike tissue-based genomic profiling, ctDNA analysis enables minimally invasive and dynamic characterization of tumor genomic landscape, potentially capturing complementary information on intratumoral heterogeneity and molecular changes during disease evolution [58]. In GEP-NENs, ctDNA currently represents a promising biomarker for prognostic assessment and disease monitoring rather than a stand-alone diagnostic tool.

The biological heterogeneity of these tumors, together with the generally low ctDNA shedding observed in well-differentiated neoplasms, limits its sensitivity for initial diagnosis and does not currently support replacing conventional tissue biopsy [58]. In contrast, ctDNA detection has been associated with increased tumor burden and aggressive disease in high-grade GEP-NENs, particularly NECs, in which ctDNA may capture molecular alterations involving genes such as *TP53* and *KRAS* [59]. In metastatic well-differentiated GEP-NETs, preliminary evidence from personalized, tumor-informed assays suggests that detectable ctDNA levels may correlate with higher tumor burden and more advanced disease [60]. These findings support the potential prognostic relevance of ctDNA, although the available evidence remains limited by small sample sizes and heterogeneous study populations.

A further potential application of ctDNA is longitudinal disease monitoring. Serial measurements allow dynamic changes in tumor-derived DNA to be evaluated during treatment and may provide an early indication of changes in disease burden. In a recent retrospective series of four patients with metastatic GEP-NETs treated with PRRT, tumor-informed ctDNA monitoring showed concordant changes with radiological disease status and treatment response, whereas CgA levels showed less consistent associations with clinical outcomes [61]. In some patients, changes in ctDNA preceded radiological evidence of disease progression, suggesting its potential utility as an early molecular indicator of treatment failure. Although these findings provide proof-of-concept for longitudinal ctDNA monitoring during PRRT, they remain preliminary and require confirmation in larger prospective cohorts.

Several limitations currently restrict the clinical translation of ctDNA in GEP-NENs. The low tumor shedding characteristic of many well-differentiated tumors can reduce detection sensitivity, while differences in assay design, sequencing strategies, variant interpretation, and analytical thresholds may contribute to variability across studies [49] (Table 2). Furthermore, much of the available evidence derives from retrospective studies, small cohorts, or preliminary reports, limiting the generalizability of the findings. In this context, multianalyte blood-based transcriptomic assays such as the NETest have shown diagnostic accuracy above 90% across in selected cohorts, although these estimates vary according to tumor stage, grade, comparator population, and assay cut-off, making their routine clinical application investigational [62]. Thus, the available evidence for ctDNA currently supports biological and clinical validity, but not yet established clinical utility. Further validation in larger, prospective, multicenter studies is needed to determine whether ctDNA-guided monitoring can improve clinical decision-making and patient outcomes and thereby support its integration into routine clinical practice.

### 4.3. MicroRNAs (miRNAs)

microRNAs (miRNAs) are small non-coding RNA molecules of approximately 20–22 nucleotides in length on average, that regulate gene expression at the post-transcriptional level and may function as oncogenes or tumor suppressors depending on the cellular context [63]. miRNAs can be detected in both solid tissues and body fluids, including plasma, serum, saliva, urine or cerebrospinal fluid, where their relative stability and accessibility support their potential use as minimally invasive biomarkers [64]. Dysregulation of miRNA expression has been described across several malignancies, where these molecules have been implicated in tumor progression, metastases, angiogenesis and metabolic reprogramming [65].

In GEP-NENs the role of circulating miRNAs remains incompletely characterized. Several studies have investigated circulating miRNA profiles as potential biomarkers for tumor detection and characterization. In an analysis of more than 700 circulating miRNAs, miR-1290 showed the highest discriminatory performance for distinguishing pancreatic ductal adenocarcinoma (PDAC) from PanNETs, with an AUC (Area Under the Curve) of 0.80 (95%; CI -confidence interval-, 0.67–0.93), whereas other candidates, including miR-628-3p, miR-550, and miR-1825, showed statistically significant but less pronounced differences [66]. However, these findings should be interpreted cautiously, as the study was primarily designed to distinguish pancreatic cancer from PanNETs and therefore does not establish miR-1290 as a diagnostic biomarker for GEP-NENs. More recently, a panel of 13 circulating miRNAs was found to be overexpressed in patients with GEP-NENs compared to healthy subjects [67]. In another study a combination of four miRNA (miR-125b-5p, miR-362-5p, miR- 425-5p and miR-500a-5p) was able to distinguish SI-NETs from healthy controls, achieving an AUC of 0.951 [68]. In GEP-NETs, circulating miRNAs have also been investigated in relation to tumor aggressiveness and PRRT response. For example, circulating miR-5096 has been associated with ^18F-FDG PET/CT positivity and SSTR2 expression in PanNETs, providing a potential mechanistic link with PRRT response [69]. These findings suggest that multi-miRNA signatures may provide more informative diagnostic profiles than individual miRNAs, although their reproducibility across independent populations remains to be established. Whether treatment-related factors, including somatostatin analogue therapy, influence circulating miRNA levels also remains unclear. Computational approaches may further enhance the diagnostic potential of multi-miRNA profiles by integrating multiple molecular signals and identifying disease-associated patterns that may not be captured by single-analyte approaches. However, evidence for machine-learning (ML)-based classification using circulating miRNAs in GEP-NENs remains limited, and currently available data are insufficient to establish such approaches as clinically validated diagnostic tools [70].

Despite these encouraging findings, the clinical translation of circulating miRNA-based biomarkers in GEP-NENs remains challenging (Table 2). Major limitations include pre-analytical and analytical variability, the lack of standardized workflows for blood collection, RNA isolation, normalization, and data analysis, relatively small study populations, and the limited reproducibility of miRNA signatures across independent cohorts. Adoption of standardized pre-analytical workflows and reporting standards, including MIQE recommendations for PCR-based assays and consensus procedures for extracellular RNA studies [71], together with appropriate exogenous controls, may improve reproducibility and facilitate independent validation of circulating miRNA signatures. In addition, circulating miRNA levels may be influenced by tumor site, grade, disease stage, and non-tumor-related biological factors, further complicating the identification of robust disease-specific signatures [65]. Thus, current evidence supports the biological relevance and potential clinical validity of circulating miRNAs, but their clinical utility has not yet been established. Further methodological standardization and prospective, multicenter validation are required to determine whether circulating miRNA-based assays can provide reproducible and clinically actionable information before their incorporation into routine clinical practice.

### 4.4. Extracellular Vesicles

Extracellular vesicles (EVs) are membrane-bound particles released by cells into the extracellular environment and biological fluids and represent an emerging component of liquid biopsy. EVs contain a complex molecular cargo, including proteins, lipids, messenger RNAs (mRNAs), miRNAs, and other nucleic acids, which may reflect biological features of their cells of origin [72], with potential diagnostic and therapeutic applications in oncology [73]. Through the transfer of their molecular cargo to recipient cells, EVs participate in intercellular communication and may contribute to tumor-related processes, including proliferation, angiogenesis, immune modulation, and metastatic dissemination [74]. According to the MISEV2023 (Minimal Information for Studies of Extracellular Vesicles 2023) recommendations, the generic term “extracellular vesicles” should be preferred unless a specific vesicle subtype can be reliably defined [75]. In particular, the term “exosome” should not be assigned solely on the basis of vesicle size or isolation procedure, but should preferably be reserved for vesicles for which an endosomal origin can be demonstrated [75]. This distinction is particularly relevant in biomarker studies, as many published analyses isolate heterogeneous small-EV populations without establishing their specific biogenesis.

In GEP-NENs, the investigation of EV-derived biomarkers remains at an early stage. Emerging evidence suggests that circulating EV populations and their molecular cargo may provide complementary information on neuroendocrine tumor biology [49]. In particular, EV-associated miRNAs and proteins have been investigated as potential biomarkers of tumor presence and biological aggressiveness, although the available evidence remains limited and heterogeneous [49]. However, many studies of circulating miRNAs in GEP-NENs do not establish whether the detected molecules are specifically associated with EVs or with other circulating compartments. Therefore, these findings support the potential relevance of circulating molecular cargo but should not be interpreted as evidence specifically attributable to exosomes or other defined EV subtypes [76]. Beyond diagnostic applications, EVs may provide a biologically informative compartment for investigating tumor–microenvironment interactions and treatment-related molecular changes. Their molecular cargo may change during tumor progression or in response to therapy and could potentially contribute to the study of mechanisms underlying therapeutic resistance [77]. However, these findings should be considered mechanistic and hypothesis-generating rather than evidence of a clinically validated exosome-based biomarker of treatment resistance. More broadly, evidence supporting the use of EV-derived biomarkers for treatment monitoring, prediction of therapeutic response, or identification of treatment resistance in GEP-NENs remains exploratory (Table 2).

A potential advantage of EV-based liquid biopsy is that the vesicular membrane may protect molecular cargo from extracellular degradation, facilitating the detection of tumor-associated RNA and proteins in circulation [72]. Conversely, the biological heterogeneity of EV populations and the frequent co-isolation of non-vesicular extracellular particles and contaminants represent major methodological challenges. MISEV2023 emphasizes the importance of standardized procedures for sample collection and pre-processing, EV separation, characterization, and reporting to improve analytical reproducibility and facilitate comparison across studies [75]. These methodological issues are particularly relevant when attempting to attribute a biomarker signal to a specific EV subtype.

Despite their biological and translational potential, the clinical utility of EV-derived biomarkers in GEP-NENs has not been established. Current evidence is largely derived from small exploratory studies, heterogeneous analytical approaches, and analysis of EV populations that cannot always be unequivocally classified according to their biogenesis [49,78]. Accordingly, current evidence supports the biological relevance and potential clinical validity of EV-based biomarkers but not yet their clinical utility. Further methodological standardization and prospective, multicenter validation are required to determine whether EV-based assays can provide reproducible and clinically actionable information before their integration into routine GEP-NEN management.

## 5. Multianalyte Approaches: NETest

NETest is a multianalyte blood-based assay that measures the expression of 51 neuroendocrine tumor-associated transcripts and generates a proprietary algorithm-based score ranging from 0% to 100% [79]. Although NETest has demonstrated high diagnostic sensitivity in selected cohorts and has been investigated as a longitudinal marker of disease activity, these performance characteristics should not be equated with clinical utility. To date, prospective evidence demonstrating that NETest-guided clinical decision-making improves treatment selection, disease control, quality of life, or survival remains lacking, and current guidelines (2023 NANETS-North American Neuroendocrine Tumor Society-and 2024 ENETS) do not currently support NETest a treatment-informing biomarker [35,80]. In a validation study including 46 patients with metastatic well-differentiated NETs, 21 patients with other metastatic gastrointestinal cancers, and 26 healthy individuals, NETest sensitivity was 98% using an upper limit of normal of 13% [81]. Specificity was 81% among healthy individuals but only 48% among patients with other gastrointestinal malignancies; using an updated upper limit of normal of 20%, specificity increased to 100% in healthy individuals and 67% in patients with other cancers [81]. These findings indicate that the high diagnostic sensitivity of NETest was demonstrated primarily in a population with metastatic, well-differentiated NETs, whereas specificity was strongly dependent on the comparator population. In a separate prospective cohort of 258 patients with NENs, including 215 with GEP-NENs, NETest was positive in 122 of 123 patients with image-positive disease (99%), substantially exceeding the detection rates obtained with the two CgA assays evaluated in the study [82]. The initial validation reported by Modlin et al. included a validation set of 40 patients with NETs and controls and a prospectively collected cohort of 41 patients with NETs, of whom 61% had SI-NETs and 50% had metastatic disease; in the prospective cohort, NETest sensitivity and specificity were both 92.8% [83]. In a cohort of 132 patients with GEP-NETs, serial NETest measurements were associated with disease course, while higher pretreatment scores were observed in patients subsequently achieving prolonged disease control. However, the predictive performance for treatment response was moderate (AUROC 0.73), and the clinical utility of serial NETest monitoring remains to be established [84]. However, these performance estimates should be interpreted in the context of the selected study populations and should be distinguished from independent validation and, importantly, from evidence demonstrating clinical utility.

Beyond diagnostic assessment, NETest has been investigated as a marker of biological disease activity and longitudinal disease status. Changes in NETest scores have been reported after surgical resection and during treatment, while rising scores have been associated with disease progression and, in selected studies, with treatment response or non-response [9]. These findings suggest potential prognostic and monitoring value. However, the evidence remains heterogeneous, with differences in tumor site, grade, treatment setting, endpoints, and NETest cut-off values across studies. Importantly, associations between NETest dynamics and radiological or clinical outcomes do not demonstrate that NETest-guided management improves patient outcomes. To date, prospective evidence showing that incorporation of NETest into clinical decision-making improves patient outcomes, including disease control, quality of life, or survival, remains lacking. More recent evidence in high-grade GEP-NENs is also consistent with a predominantly prognostic rather than predictive role, as elevated NETest levels were associated with shorter overall survival but baseline NETest did not predict benefit from chemotherapy [85].

This distinction is reflected in current clinical guidance. The 2023 NANETS guidelines report that higher NETest scores may have prognostic value and that changes in NETest have been associated with treatment response in selected studies; however, the overall quality of evidence is very low, and it remains unclear whether NETest-based monitoring improves quality of life or clinical outcomes compared with conventional imaging [80]. The guidelines therefore conclude that NETest may have prognostic value but is not currently a treatment-informing biomarker. Similarly, the 2024 ENETS guidance for well-differentiated SI-NETs acknowledges that NETest may be considered for treatment monitoring, while emphasizing that its clinical usefulness remains under evaluation and that the test is not widely available [35] (Table 2).

Additional barriers to broader implementation include the cost of testing, limited availability, and the need for specialized laboratory infrastructure and expertise [62]. These factors are particularly relevant because high analytical or diagnostic performance does not, by itself, establish clinical utility. Biomarker-guided testing must also be reproducible and demonstrate that its incorporation into clinical decision-making improves patient management or outcomes. Taken together, current evidence supports NETest as a potentially informative prognostic and monitoring biomarker, but its clinical utility remains unproven.

## 6. Tissue/Molecular Biomarkers: Spatial Transcriptomics as a Discovery Platform

Recent advances in spatially resolved molecular technologies have introduced spatial transcriptomics as a promising approach for investigating intratumoral heterogeneity in GEP-NENs while preserving tissue architecture [86]. Unlike bulk transcriptomic analyses, which average molecular signals across heterogeneous tumor and microenvironmental populations, spatial transcriptomics enables transcriptional programs to be examined within their anatomical context [86]. This may be particularly relevant in GEP-NENs, in which differences in cellular state, tumor–microenvironment interactions, and spatial organization may contribute to biological behavior beyond that captured by conventional histopathology and bulk molecular profiling [8,87]. Given the relatively low mutational burden observed in many GEP-NENs, transcriptional regulation and cellular plasticity may represent important determinants of tumor phenotype beyond genomic alterations alone. In this context, spatial transcriptomics may complement genomic profiling by identifying biologically relevant cellular states that are not detectable through DNA-based analyses.

Although studies specifically addressing GEP-NENs remain limited, emerging evidence suggests that spatially resolved molecular profiling may identify distinct tumor cellular states and microenvironmental compartments [87]. Preliminary evidence from a recent multi-omic pre-print including 38 well-differentiated pancreatic and SI-NETs showed that spatial transcriptomic analysis identified distinct transcriptional programs associated with immune-rich, stromal, and vascularized tumor niches, including enrichment of one program in metastatic lesions [87]. In another study, spatial transcriptomics was performed on 72 tissue microarray cores derived from four patients with multifocal SI-NETs, including tumoral and non-tumoral tissues. The study revealed four distinct tumor subtypes associated with their anatomical location—mucosal, mesenteric, lymphatic, and deep—with distinct transcriptional profiles and ligand–receptor interactions, suggesting that local interactions with the microenvironment may contribute to tumor phenotypic heterogeneity [88]. Spatial profiling studies in GEP-NETs have revealed tissue-specific neuro-immune interactions, highlighting the contribution of the local microenvironment to neuroendocrine differentiation and tumorigenesis [89]. These findings suggest that spatially resolved approaches may provide additional information on intratumoral heterogeneity, tumor–stroma interactions, immune organization, and the molecular organization of regional metastatic sites. However, the available evidence remains preliminary, with small cohorts and limited independent validation.

From a translational perspective, spatial transcriptomics may complement genomic and conventional transcriptomic profiling by identifying biologically relevant cellular states that are obscured by bulk analyses [86]. Integration with single-cell transcriptomics, proteomics, and digital pathology may further facilitate the characterization of GEP-NEN heterogeneity and the identification of potential candidate biomarkers for biological classification, prognostic assessment, and therapeutic stratification [8,87]. Nevertheless, these applications remain investigational, and there is currently insufficient evidence to determine whether spatially resolved molecular profiles provide clinically relevant information beyond established histopathological, imaging, and molecular parameters.

The clinical translation of spatial transcriptomics in GEP-NENs is currently limited by high costs, technical complexity, limited tissue availability, platform-dependent resolution, and the absence of standardized analytical pipelines [90]. Most GEP-NEN-specific studies remain exploratory, with limited cohort sizes and insufficient prospective or independent validation. Therefore, spatial transcriptomics should currently be regarded primarily as a discovery and translational research platform rather than a clinically validated diagnostic or treatment-selection tool (Table 3).

## 7. Molecular Imaging and Radiomics-Based Biomarkers

Molecular imaging provides biologically relevant information that complements conventional anatomical imaging and histopathological assessment in GEP-NENs, particularly by enabling non-invasive characterization of SSTR expression and glucose metabolism [91,92]. SSTR-PET (Positron Emission Tomography)/CT (Computed Tomography) has become a standard imaging modality for the staging and characterization of well-differentiated NETs and can provide information directly linked to a therapeutically relevant molecular target [91,92]. In particular, radiolabeled somatostatin analogs such as ^68^Ga-DOTATATE enable the detection of SSTR-expressing primary and metastatic lesions and contribute to the identification of patients potentially eligible for PRRT [93]. SSTR-PET can therefore be regarded not only as a staging modality but also as a whole-body molecular imaging phenotype reflecting the spatial distribution of a SSTR expression [94]. However, imaging-based assessment of SSTR expression should be considered complementary to tissue-based characterization, as SSTR-PET uptake may not always correspond to tissue-level receptor expression, as shown in lung NETs [95].

In contrast ^18^F-fluorodeoxyglucose (^18^F-FDG) PET/CT provides information on tumor glucose metabolism, and is generally associated with high-grade, more aggressive tumor phenotype and poorer clinical outcomes [96]. Its prognostic relevance is well established, whereas its impact on treatment decision-making remains more heterogeneous and context-dependent [97]. The complementary use of SSTR-PET and ^18F-FDG PET/CT, commonly referred to as dual-tracer imaging, may provide a more comprehensive characterization of tumor biology and reveal interlesional and intralesional heterogeneity [98]. Discordant uptake patterns may include lesions with preserved SSTR expression but increased glycolytic activity, as well as SSTR-negative and metabolically active tumor components, potentially highlighting areas of biological aggressiveness that may not be fully captured by conventional histopathological grading [98,99]. Several studies have reported associations between dual-tracer imaging patterns and clinical outcomes, supporting their potential relevance for biological risk characterization and clinical management [98,99]. Nevertheless, these imaging phenotypes should currently be regarded as complementary biological information rather than independently validated molecular biomarkers, and prospective evidence is still required to establish whether their integration into treatment decision-making improves patient outcomes (Table 3).

Radiomics represents a quantitative imaging approach that enables the extraction of high-dimensional features from routinely acquired clinical images [100,101,102]. These features can capture tumor shape, intensity, texture, and spatial heterogeneity and can subsequently be analyzed using statistical or machine-learning (ML) approaches to identify quantitative patterns that may not be readily recognized by conventional visual assessment [103]. In GEP-NENs, radiomic features have been investigated using CT, MRI (Magnetic Resonance Imaging), and PET for tumor characterization, grading, assessment of lymph-node involvement, treatment response, and prediction of clinical outcomes. A systematic review of GEP-NET radiomics identified promising associations with tumor grade and differential diagnosis but also highlighted substantial methodological limitations, including small retrospective cohorts, heterogeneous image acquisition and reconstruction protocols, variability in segmentation and feature extraction, and limited external validation [104]. More recent studies have extended radiomics toward clinically relevant endpoints, including the identification of imaging features associated with treatment response to somatostatin analogs and the assessment of lymph-node metastases using ^68Ga-DOTATOC PET radiomics [105,106]. These findings support the potential of radiomics to extract complementary quantitative information from both anatomical and molecular imaging; however, current evidence remains predominantly exploratory.

**Figure 2:**
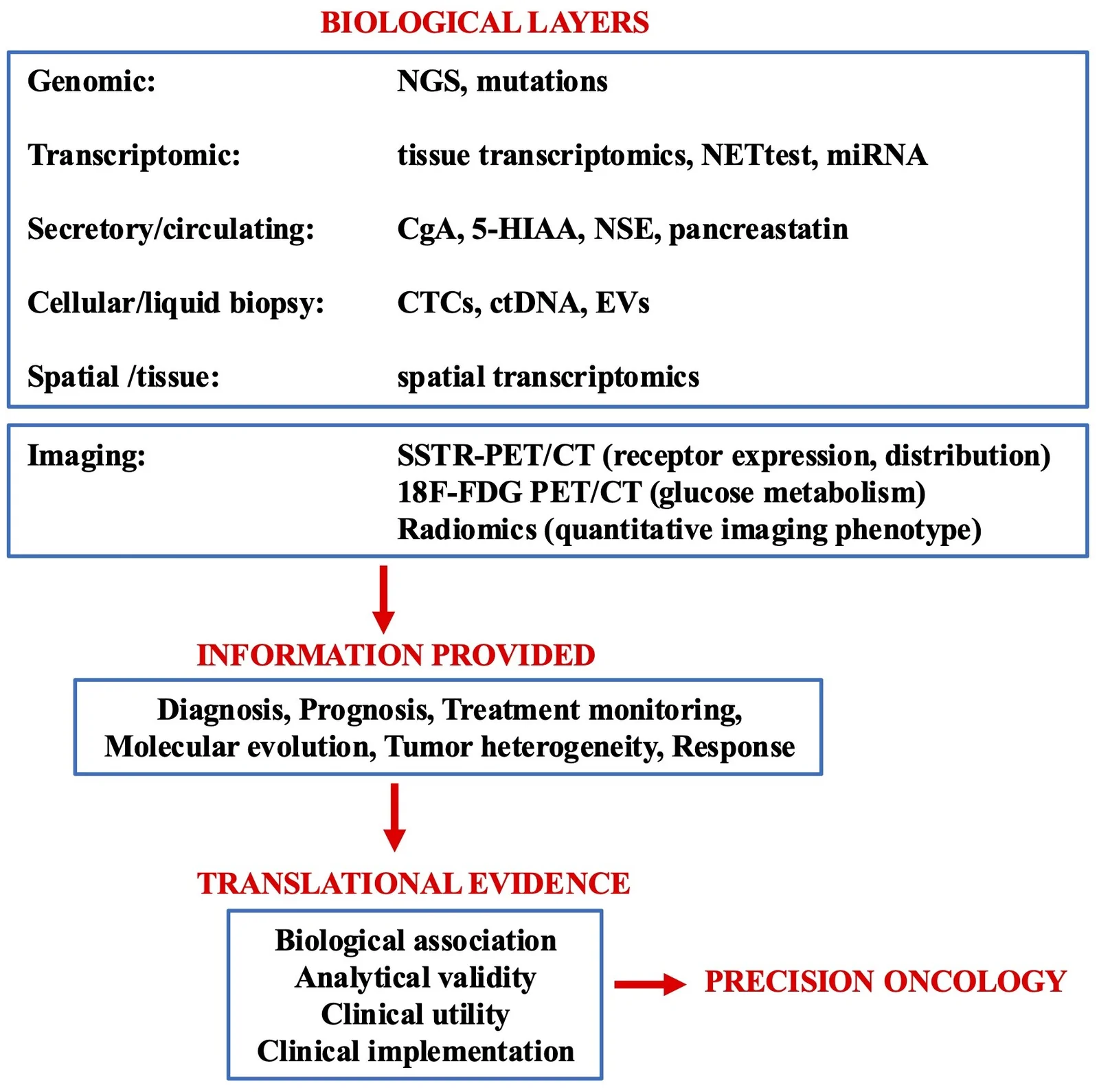
Complementary biological layers and their potential integration in GEP-NEN precision oncology.

From a biomarker-development perspective, radiomic signatures should therefore be distinguished from validated clinical biomarkers. Associations between radiomic features and tumor characteristics or clinical outcomes demonstrate biological or clinical validity but do not establish analytical robustness, clinical utility, or incremental value over established clinicopathological and imaging parameters [104]. The translational potential of radiomics may be enhanced by integrating imaging-derived features with genomic, transcriptomic, circulating, and clinical data. Such radiogenomic and multimodal approaches may enable more comprehensive characterization of tumor heterogeneity and disease evolution, although their clinical applications remain investigational in GEP-NENs [107,108]. However, evidence derived from other tumor types cannot be directly extrapolated to GEP-NENs, and methodological heterogeneity remains a major barrier to clinical translation. Differences in image acquisition and reconstruction, lesion segmentation, preprocessing, feature extraction, feature harmonization, and model development may substantially affect reproducibility and generalizability. Accordingly, radiomics and radiogenomics should currently be regarded as emerging approaches for biomarker development in GEP-NENs, rather than established clinical tools, given the limited external validation and methodological heterogeneity of the available evidence [103,104,109]. In Figure 2 complementary biological information provided by biomarker and imaging approaches in GEP-NENs are shown.

## 8. Artificial Intelligence (AI) AI and Multimodal Integration

The limitations of single-biomarker approaches have stimulated a transition from isolated analytes toward integrated strategies combining complementary molecular, cellular, imaging, and clinical information [110]. In GEP-NENs, however, multimodal integration should not be understood simply as the parallel availability of multiple molecular or imaging technologies. Rather, meaningful integration requires the combination of biologically complementary data layers to address a defined biological or translational question. This distinction is particularly relevant in GEP-NENs because the different biomarker classes described above interrogate distinct dimensions of tumor biology rather than interchangeable representations of the same process.

Genomic profiling provides information on relatively stable molecular alterations, including mutations and copy-number changes identified through next-generation sequencing (NGS), whereas transcriptomic approaches capture dynamic cellular states and patterns of gene expression [13,111]. Protein-based and secretory biomarkers, such as CgA and pancreastatin, provide information related to the neuroendocrine secretory phenotype [22], while ctDNA and CTCs may provide minimally invasive information on tumor burden and molecular evolution [49,112,113]. Molecular imaging provides complementary whole-body and spatial information, including the distribution of SSTR expression and glucose metabolism, while radiomics may quantify imaging phenotypes and spatial heterogeneity that are not readily captured by visual assessment [92,103,104]. Spatial transcriptomics further connects molecular states with tissue architecture and may help characterize the relationship between tumor cells and their microenvironment [86,87]. Thus, these approaches should be regarded as complementary biological layers rather than interchangeable biomarkers.

Artificial intelligence (AI) and ML methods provide computational frameworks for integrating high-dimensional and heterogeneous datasets [114,115]. By integrating high-dimensional molecular, histopathological, imaging, and clinical variables, AI-based approaches may support tumor classification and prognostic stratification in selected research settings, although evidence in GEP-NENs remains limited and predominantly exploratory. Broader evidence from other malignancies should therefore be regarded as methodological context rather than direct evidence of clinical validity in GEP-NENs [116,117]. A recent study developed and independently validated a ML classifier using peripheral blood RNA-seq for non-invasive detection of GEP-NETs and assessment of treatment response to 177Lu-DOTATATE PRRT, highlighting its potential while underscoring the need for further validation before clinical implementation [118].

GEP-NENs represent a particularly challenging setting for AI-based models because of their relative rarity, marked biological heterogeneity, and differences in clinical presentation, tumor site, differentiation, grade, and treatment exposure. Small training cohorts increase the risk of overfitting, while differences in sequencing platforms, sample processing, imaging acquisition and reconstruction, pathology workflows, feature extraction, and patient populations may impair external generalizability [116,117,119]. Integration of multiple data types also introduces challenges related to missing data, differences in scale and dimensionality, temporal alignment, and harmonization of analytical pipelines. High predictive performance in an internal dataset therefore does not establish biological validity, clinical validity, or clinical utility. The translational value of AI-based models will depend on reproducibility, calibration, external validation, and demonstration of incremental value over established clinicopathological and imaging parameters [119,120]. Deep-learning and other complex models may also be difficult to interpret, creating a potential black-box problem when molecular, imaging, or clinical predictions are translated into biological mechanisms or treatment recommendations [121].

Overall, the development of precision biomarkers in GEP-NENs is unlikely to depend on the identification of a single superior analyte. Rather, future progress will require biologically informed combinations of genomic, transcriptomic, circulating, spatial, histopathological, imaging, and clinical information, with AI serving as an analytical framework for their integration. Such approaches may improve the characterization of tumor heterogeneity and evolution, but their clinical role should remain primarily investigational until their reproducibility, biological validity, external generalizability, and clinical utility are established.

## 9. Conclusions and Future Perspectives

GEP-NENs are biologically and clinically heterogeneous neoplasms, and no single biomarker currently provides a comprehensive representation of tumor biology, disease evolution, and treatment response. This review moves beyond a descriptive classification of GEP-NEN biomarkers by framing their development along a translational continuum from biological signal and clinical validity to demonstrated clinical utility and potential implementation. By considering conventional biomarkers together with liquid biopsy, multianalyte, spatial, imaging, and AI-based approaches, this framework highlights that the major unmet need is no longer simply the identification of biomarkers with diagnostic or prognostic associations, but the demonstration that these signals provide reproducible, incremental, and clinically actionable information. This perspective places clinical utility as the critical interface between biomarker discovery and precision oncology and provides a rationale for future prospective studies designed to validate multimodal biomarker strategies. A summary of the biological layers, analytical approaches, evidence levels, major limitations, and current translational status of the strategies discussed in this review is provided in Table 4.

Established circulating biomarkers, such as CgA and 5-HIAA, remain clinically useful in selected settings, but their interpretation is highly dependent on tumor site, functional phenotype, disease burden, and clinical context. Their limitations illustrate the broader challenge of applying single-analyte biomarkers to a heterogeneous disease spectrum. Emerging approaches are progressively expanding the biological information available from GEP-NENs. Liquid biopsy strategies, including CTCs, ctDNA, circulating miRNAs, and extracellular vesicles, may provide minimally invasive and longitudinal information on tumor burden, molecular alterations, and disease evolution. Multianalyte transcriptomic assays such as NETest may provide additional information on disease activity and prognosis, although their clinical utility and treatment-informing value remain to be established. Similarly, spatial transcriptomics, radiomics, and other quantitative imaging approaches may provide complementary information on intratumoral heterogeneity, tissue organization, and whole-body tumor phenotype, but remain predominantly investigational.

A central distinction emerging from the available evidence is that biological association and diagnostic or prognostic performance do not necessarily translate into clinical utility. Across the biomarker landscape, many approaches have demonstrated promising analytical or clinical validity, whereas prospective evidence showing that biomarker-guided strategies improve treatment selection, clinical decision-making, quality of life, or survival remains limited. This distinction should remain central to the interpretation and future development of GEP-NEN biomarkers.

Future research should therefore move beyond the identification of individual candidate biomarkers toward the development and validation of biologically informed multimodal strategies. Integration of genomic, transcriptomic, circulating, spatial, histopathological, molecular imaging, and clinical information may provide a more comprehensive representation of tumor heterogeneity and temporal evolution. AI and machine-learning methods may serve as computational frameworks for integrating these complementary data layers; however, their clinical translation will require rigorous external validation, reproducibility, calibration, interpretability, and demonstration of incremental clinical utility over established clinicopathological and imaging parameters.

The most promising future directions are therefore not the replacement of conventional pathology, imaging, or established biomarkers by a single novel technology, but their integration into validated, clinically actionable frameworks. Prospective multicenter studies, standardized analytical workflows, independent validation, and appropriate health-economic evaluation will be essential to determine which emerging biomarkers can progress from biological discovery to clinically meaningful applications. Until such evidence is available, most liquid biopsy, spatial, radiomic, and AI-based approaches should be considered complementary investigational tools rather than substitutes for established clinical assessment. Ultimately, precision oncology in GEP-NENs will depend on identifying biomarker strategies that provide information beyond existing clinical, pathological, and imaging parameters and, most importantly, demonstrate that this additional information leads to better clinical decisions and improved patient outcomes.

## Figures and Tables

**Table 1:** Classical circulating biomarkers in GEP-NENs: clinical applications, limitations, and level of evidence.

Biomarker Approach	Main Clinical Question	Main Population/ Tumor Site	Diagnostic Performance	Prognostic Value	Predictive Value	Evidence Base	Main Limitation	Guideline Status
CgA	Diagnosis/ treatment monitoring	Mainly wd-NETs; context/ site dependent	Moderate/ variable	Moderate	Not established	Prospective/retrospective; multiple cohorts	PPI use; renal dysfunction; assay variability	Selected clinical contexts; not recommended for screening
5-HIAA	CS diagnosis; treatment monitoring	Serotonin-secreting SI-NET/CS	High in CS; lower without CS	Limited/ moderate	Not established	Prospective/retrospective	Diet: malabsorption; limited applicability	Routine when clinically indicated; suspected/established CS
NSE	Prognosis/ treatment monitoring	High-grade NENs, particularly NEC	Limited	Potential	Not established	Retrospective; exploratory prospective	Low specificity	High-grade NENs, particularly NEC/NET G3
Pancreastatin	Prognosis	Small bowel NETs/PanNETs	Not established	Not established	Not established	Retrospective; limited cohorts	Insufficient evidence	Not established

wd-NETs: well-differentiated NETs; PPI: proton pump inhibitor; CS: carcinoid syndrome; SI-NETs: small intestinal-NETs.

**Table 2:** Emerging circulating and multianalyte biomarker approaches in GEP-NENs: clinical applications, evidence, limitations, and translational status.

Biomarker Approach	Main Clinical Question	Main Population/ Tumor Site	Diagnostic Performance	Prognostic Value	Predictive Value	Evidence Base	Main Limitation	Guideline Status
CTCs	Prognosis/treatmentmonitoring	Metastatic NET	Limited sensitivity	Potential	Not established	Prospective/retrospective	Low Detection rate; platform dependence	Investigational
ctDNA	Prognosis/ Molecular monitoring	Mainly advanced/ high-grade disease	Limited in low-shedding tumors	Promising	Not established	Mostly retrospective/small prospective studies	Low shedding, assay heterogeneity	Investigational
miRNAs	Diagnosis/prognosis	Site- dependent	Exploratory/ promising	Exploratory	Not established	Mostly retrospective	Preanalytical and analytical variability; limited reproducibility	Investigational
EVs	Molecular characterization	Exploratory	Not established	Exploratory	Not established	Early/preclinical	Isolation and characterization	Investigational
NETest	Diagnosis/treatmentmonitoring	Mainly metastatic wd-SI-NETs	High in selected cohorts	Potential	Not established	Prospective/retrospective; limited independent validation	Availability; cost; standardization; unproven clinical utility	Investigational; not currently treat ment-informing

wd-SI-NET: well-differentiates small intestinal-NETs.

**Table 3:** Emerging imaging, spatial, and computational approaches in GEP-NENs: current evidence, clinical relevance, and translational limitations.

Biomarker Approach	Main Clinical Question	Population Site	Diagnostic Performance	Prognostic Value	Predictive/Theranostic Relevance	Evidence Base	Main Limitation	Guideline Status
Molecular imaging (SSTR-PET/FDG-PET)	Diagnosis/ Staging/ Prognosis/theranostic selection	Mainly wd-NETs	Established	Established	Context-dependent	Prospective/ retrospective	Biological heterogeneity; limited standardization of quantitative metrics	Routine clinical use
Radiomics/radiogenomics	Diagnosis/prognosis/response assessment	Site- and imaging-dependent	Promising	Promising	Not established	Mostly retrospective	Overfitting, methodological heterogeneity; limited external validation	Research only
Spatial transcriptomics	Discovery/ Heterogeneity cgaracterization	Tissue-based	Not established	Exploratory	Not established	Small exploratory cohorts	Cost, tissue requirements; technical complexity; lack of standardization	Research only
AI/multimodal models	Classification/ prognosis/response assessment	Heterogeneous	Promising	Exploratory	Not established	Mostly retrospective/exploratory	Small datasets; overfitting; limited generalizability; interpretability	Research only

**Table 4:** Integrated biomarker landscape in GEP-NENs: biological layers, analytical approaches, evidence, and translational challenges.

Biological Layer	Specimen/Data Source	Biomarker/Approach	Biological Information Captured	Evidence Level	Major Limitations	Translational Status
Histopathology	Tissue	Morphology, Ki-67, IHC	Differentiation, proliferation, phenotype	Established	Sampling bias, heterogeneity	Routine
Genomic	Tissue	NGS, *MEN1*, *DAXX*, *ATRX*, *TP53*, *RB1*	Genomic alterations, molecular phenotype	Established/for selected applications; exploratory for biomarker-guided use	Site- and grade-dependence; limited predictive value	Selected/investigational
Circulating protein	Blood	CgA, 5-HIAA, NSE, pancreastatin	Secretory phenotype, biochemical activity	Established in selected settings	Low specificity; context dependence	Clinical use in selected settings
Cellular liquid biopsy	Blood	CTCs	Tumor dissemination, cellular phenotype	Promising; limited validation	Low detection; platform dependence	Investigational
Circulating DNA	Blood	ctDNA	Tumor-derived genomic alterations, molecular evolution	Promising; limited clinical validation	Low shedding; assay heterogeneity	Investigational
Circulating RNA	Blood	miRNAs	Regulatory/transcriptional signatures	Exploratory	Pre-analytical variability; limited reproducibility	Investigational
Extracellular vesicles	Blood/ body luids	EV-associated RNA/proteins	Intercellular signaling, tumor biology	Exploratory	Isolation/characterization	Research
Multianalyte transcriptomic	Blood	NETest	Composite transcriptional disease signal	Promising; clinical utility unproven	Standardization, cost, clinical utility	Investigational
Molecular imaging	Whole body	SSTR-PET, FDG-PET	Receptor phenotype, metabolism, spatial heterogeneity	Established	Biological heterogeneity; quantitative standardization	Clinical use
Quantitative imaging	Imaging	Radiomics	Imaging phenotype, heterogeneity	Exploratory/promising preliminary evidence	Overfitting; external validation	Research
Radiogenomics	Imaging plus tissue/molecular data	Imaging-genomic integration	Imaging phenotype linked to molecular/genomic characteristics	Exploratory	Methodologial heterogeneity; limited external validation	research
Spatial transcriptomics	Tissue	Spatial gene-expression profiles	Cellular states, tissue architecture, microenvironment	Exploratory	Cost, tissue, standardization	Research
Multimodal computational	Multiple data layers	AI/ML	Integrated molecular, imaging, and clinical patterns	Exploratory	Small datasets, overfitting, Interpretability, generalizability	Research

## Data Availability

No new data were created or analyzed in this study. Data sharing is not applicable to this article.

## Abbreviations

The following abbreviations are used in this manuscript:

5-HIAA	5-hydroxyindoleacetic acid
^18^F-FDG	^18^F-fluorodeoxyglucose
AI	artificial intelligence
ALT	alternative lengthening of telomeres
ATRX	alpha-thalassemia/mental retardation syndrome X-linked
AUC	Area Under the Curve
CgA	chromogranin A
ctDNA	circulating tumor DNA
CD56	cluster of differentiation 56
CI	confidence interval
CS	carcinoid syndrome
CT	Computed Tomography
CTCs	circulating Tumor Cells
DAXX	death domain-associated protein
EMT	epithelial–mesenchymal transition
ENETS	European Neuroendocrine Tumor Society
EpCAM	epithelial cell adhesion molecule
EVs	extracellular vesicles
FDA	Food and Drug Administration
GEP-NENs	gastroenteropancreatic-neuroendocrine neoplasms
GI-NETs	gastrointestinal-NETs
IHC	immunohistochemistry
MEN1	multiple endocrine neoplasia type 1
ML	machine learning
MRI	Magnetic resonance imaging
mRNAs	messenger RNAs
miRNAs	microRNAs
mTOR	mammalian target of rapamycin
NANETS	North American Neuroendocrine Tumor Society
NECs	neuroendocrine carcinomas
NENs	neuroendocrine neoplasms
NETs	neuroendocrine tumors
NGS	next-generation sequencing
NSE	neuron-specific enolase
OS	overall survival
PanNET	pancreatic neuroendocrine tumor
PDAC	pancreatic ductal adenocarcinoma
PET	Positron Emission Tomography
PPI	Proton pump inhibitor
PRRT	peptide receptor radionuclide therapy
SI-NETs	small intestinal-NETs
SSA	somatostatin analogue
SSTR	somatostatin receptors
WHO	World Health Organization
